# Conductivity and lithiophilicity gradients guide lithium deposition to mitigate short circuits

**DOI:** 10.1038/s41467-019-09932-1

**Published:** 2019-04-23

**Authors:** Jun Pu, Jiachen Li, Kai Zhang, Tao Zhang, Chaowei Li, Haixia Ma, Jia Zhu, Paul V. Braun, Jun Lu, Huigang Zhang

**Affiliations:** 1 National Laboratory of Solid State Microstructures, College of Engineering and Applied Sciences, Collaborative Innovation Center of Advanced Microstructures, and Institute of Materials Engineering, Nanjing University, Nanjing, 210093 Jiangsu China; 2 School of Chemical Engineering, Northwest University, Shaanxi 910069 Xi’an, China; 3 Key Lab of Nanodevices and Applications, Suzhou Institute of Nanotech and Nanobionics, Chinese Academy of Sciences, University of Chinese Academy of Sciences, 215000 Suzhou, China; 4 Chemical Sciences and Engineering Division, Argonne National Laboratory, Argonne, IL 60439 USA; 5 Department of Materials Science and Engineering, Frederick Seitz Materials Research Laboratory, Beckman Institute for Advanced Science and Technology, Department of Chemistry, University of Illinois at Urbana-Champaign, Urbana, IL 61801 USA

**Keywords:** Energy storage, Batteries

## Abstract

Lithium metal anodes hold great promise to enable high-energy battery systems. However, lithium dendrites at the interface between anode and separator pose risks of short circuits and fire, impeding the safe application. In contrast to conventional approaches of suppressing dendrites, here we show a deposition-regulating strategy by electrically passivating the top of a porous nickel scaffold and chemically activating the bottom of the scaffold to form conductivity/lithiophilicity gradients, whereby lithium is guided to deposit preferentially at the bottom of the anode, safely away from the separator. The resulting lithium anodes significantly reduce the probability of dendrite-induced short circuits. Crucially, excellent properties are also demonstrated at extremely high capacity (up to 40 mAh cm^−2^), high current density, and/or low temperatures (down to −15 °C), which readily induce dendrite shorts in particular. This facile and viable deposition-regulating strategy provides an approach to preferentially deposit lithium in safer positions, enabling a promising anode for next-generation lithium batteries.

## Abstract (web summary)

Here the authors report a deposition-regulating strategy to form conductivity and lithiophilicity gradients which serve to guide the preferential lithium growth away from the interface between anode and separator and mitigate the dendrite-induced short circuits.

## Introduction

Lithium (Li) metal is an attractive anode material for Li-based batteries because of its extremely high theoretical capacity and low electrode potential^1–5^. However, the non-ideal growth of Li dendrites during recharging limits its practical application^6^. During charge–discharge cycles, the repeated formation of Li dendrites may induce “dead Li” that was electrically isolated by solid electrolyte interphase (SEI), decreasing the Coulombic efficiency (CE) and usable capacity. Another major problem is that dendrite penetration of the separator induces short circuits and, in some instances, can cause fire^7–10^. Thus, dendrite-induced short circuits must be avoided for safe and reliable Li-metal anodes^11^.

Various approaches have been explored to suppress Li dendrites by modifying organic electrolyte^12^, developing solid-state electrolyte^13,14^, constructing high-modulus interfacial layers^15^, preforming artificial SEI layers^16^, preparing lithiophilic substrates^17^, and scaffolding Li^18,19^. These attempts have demonstrated very effective progress on suppressing dendrite. However, short circuits mostly related to the dendrites along the anode/separator interface instead of those at the bottom of a Li anode, indicating that the position of Li dendrites in the three-dimensional (3D) space of a Li anode severely affects the safety of Li batteries.

**Fig. 1:**
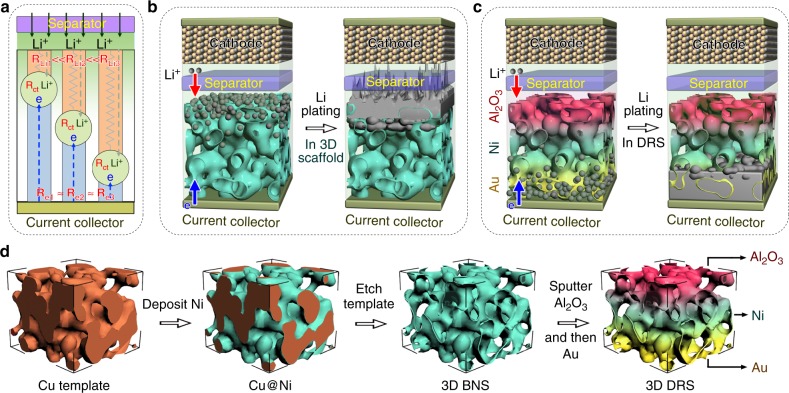
Top– and bottom–up growth modes, and the DRS fabrication route. **a** The variation of transport resistances of Li-ion and electron, and charge-transfer resistances in the through-thickness direction of a Li anode. **b** The top-growth mode at the anode/separator interface. **c** The bottom–up Li deposition in a DRS anode. **d** The DRS fabrication process

Li deposition is a diffusion-coupled reaction process. The spots and rates of Li deposition are mainly determined by multiphysical fields parameters (Li-ion concentration, local potentials, local current, etc.), which may eventually manifest themselves in terms of three simple resistances (Fig. 1a). The first is the electric resistance along the electron pathway from deposition spots to current collectors (*R*_e_), whose distribution is usually uniform because of the good electronic conductivity of the anode. The second is the Li-ion transport resistance from separator to deposition spots (*R*_Li_), which is smallest at the anode/separator interface. The third is charge-transfer resistance (*R*_ct_), which is determined by the reaction activity of solid/electrolyte interface. The smaller *R*_e_, *R*_Li_, and *R*_ct_ are, the higher the probability of Li deposition is. The basic structure of batteries, which consist of stacked cathodes, separators, and anodes, inevitably leads to nonuniform distributions in the fields of Li-ion concentration and gradients. In particular, the high Li-ion concentration, high concentration gradient, and high Li-ion flux in the anode electrolyte allow Li ions to more easily access the anode/separator interface (low *R*_Li_) during recharging. Such nonuniform fields, at severe conditions like high rates and low temperatures, usually accelerate dendrite penetration of separator and result in battery failure. Unfortunately, conventional strategies are usually not effectively to mitigate such nonuniform distributions.

The preferential deposition of Li metal at anode/separator interfaces is commonly called the “top-growth” mode (Fig. 1b), which have been revealed by many empirical observations and simulations^20–22^. Because these interfaces are closest to the counter electrodes, the top-growth mode increases the risk of short circuits. To eliminate the top-growth, we must decrease *R*_e_, *R*_Li_, and *R*_ct_ at potentially unsafe interfaces. Although it is usually impossible to reverse the increasing trend of *R*_Li_ from the separator to the anode bottom, there are plenty of spaces allowing us to tune *R*_e_ and *R*_ct_. Electric passivation can be a direct approach to suppress the top-growth of Li. We hypothesize that, in addition to suppressing the top-growth mode, promoting growth at the bottom (Fig. 1c) can further reduce the probability of unsafe dendrite formation. Recent studies have reported that Li prefers to grow on substrates (Au, Pt, Sn, ZnO, etc.) with low overpotentials or energy barriers for nucleation^23–26^. Thus, selectively applying the low barrier materials to the bottom of 3D structured anodes can be regarded as a thermodynamic strategy of lithiophilicity modification to guide Li deposition away from the anode/separator interface. Kinetic factors, such as temperature and current density, also play key roles in dendrite-induced short circuits. Because of the reduced mobility of Li ions, low temperatures readily induce the marked dendrite growth, especially at high rates/capacities, and increase the possibility of short circuits^27–31^. There is an increasing demand on high loading and concerns about low temperatures and long cycling history. At such severe conditions, the polarization along the through-thickness direction may increase to be larger than the difference of nucleation overpotentials between materials^23,32^. The preference of Li nucleation caused by using lithiophilic modification may not be high enough to tune the Li deposition. Studies on severe conditions are urgently needed to provide safer designs for Li-metal anodes. Therefore, the tunability of Li deposition must be increased greatly.

Here, we propose a deposition-regulating strategy to guide Li growth away from the unsafe anode/separator interfaces, in particular, from an overall viewpoint of tuning the series resistances of local spots according to multiphysic field distributions. The resultant deposition-regulating scaffold (DRS) can load Li up to 40 mAh cm^−2^, which is among the highest capacity of the reported metallic Li anodes. More importantly, the DRS anode also demonstrates excellent cyclability from room temperature to −15 °C, which enables the operations at harsh conditions and helps to build safer Li batteries under normal conditions. We first prepare a highly porous, bare nickel scaffold (BNS) via templated electrodeposition and selective etching (Fig. 1d). The top region of the BNS scaffold is electrically passivated by coating with alumina to prevent the top-growth mode whereas the bottom part is activated by a low-nucleation barrier Au layer to guide Li plating (Fig. 1c). The Al_2_O_3_ coating lowers the local conductivity in the top region to form a conductivity gradient as compared to the bottom metallic Ni/Au. Due to the almost-zero nucleation barrier, Au at the bottom serves as the lithiophilic coating to form a lithiophilicity gradient as compared to high-barrier Al_2_O_3_ coating. The resulting DRS anode demonstrates a high CE of ~98.1% for 500 cycles with 3.5 mAh cm^−2^ at 2 mAcm^−2^. Even at high rate (10 mAcm^−2^) and very low temperature (−15 °C), it also displayed higher CEs and better cyclability than Li anodes on Cu foil and BNS. Therefore, we have shown that our strategy is effective at circumventing unsafe interface dendritic growth caused by the intrinsic nonuniformity of multiphysical fields in conventional stacking battery structures and may facilitate the development of safer Li batteries.

## Results

### Structural characterization of DRS

**Fig. 2:**
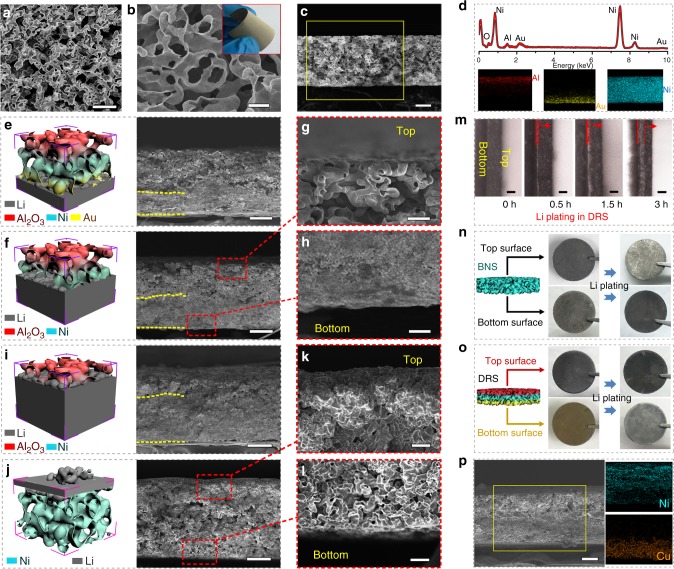
SEM and optical microscopy characterization of BNS and DRS. SEM images of the top surface of **a** BNS and **b** DRS, and of **c** the cross section of the DRS. **d** EDS plot and elemental mapping of Al, Au, and Ni in the cross section of the DRS. **e**−**l** Schematic illustration and cross-sectional SEM images of the Li-plating process: Bottom–up plating in the DRS with capacities of **e** 3, **f**−**h** 5, and **i** 8 mAh cm^−2^, respectively (the yellow dash lines indicates the edges of Li-deposited regions). **j**−**l** Surface plating in a BNS with a capacity of approximately 3 mAh cm^−2^. **m** Optical microscopy images of the cross section of the DRS, which show the Li-plating process over time (the red arrow indicates the direction of Li growth). Photographs of **n** BNS and **o** DRS, respectively, before and after Li plating. **p** SEM and EDS elemental mapping images of a Li-plated DRS, which was treated with CuCl_2_ solution in dimethoxyethane. Scale bars: (**a**, **c**, **e**, **f**, **i**, **j**, **p**), 25 μm; (**b**, **g**, **h**, **k**, **l**), 5 μm

Figure 2a, b show the scanning electron microscopy (SEM) images of the obtained BNS and DRS samples, respectively. The BNS porosity is as high as 90.4%, which provides a high volume for Li plating. All the pores of BNS are interconnected because the Cu template was annealed to form sintering necks, leading to a continuous network as shown in Supplementary Fig. 1. The interconnected pores of BNS can facilitate mass transport of Li-ions. The thickness of BNS can be readily tuned by varying the thickness of the Cu template (see Supplementary Fig. 2). Fig. 2c presents a cross-sectional SEM image of a typical DRS with the thickness of ~70 μm. As shown in the energy dispersive X-ray spectroscopy (EDX) mapping images (Fig. 2d), Al and Au were locally distributed in the top and bottom regions of the nickel scaffold, respectively. This indicates the successful fabrication of the DRS.

A capacity-limited protocol was used to plate and strip Li on the DRS and BNS (with the cell configuration illustrated in Supplementary Fig. 3). Figure 2e−i show the cross-sectional SEM images of Li-plated DRS electrodes with different capacities (3, 5, and 8 mAh cm^−2^), along with corresponding schematic illustrations of bottom–up Li plating. By carefully inspecting the cross-sectional image at the lowest capacity (Fig. 2e), one can see that the bottom of the DRS electrode was almost completely plated with solid Li. However, the top region remained porous. As the capacity increased (Fig. 2f−i), Li gradually filled up the pores, indicating a bottom–up plating mode. The bare surface on the top (Fig. 2g) and the solid Li at the bottom (Fig. 2h) indicate that Li was preferentially plated in the bottom region, where a gold layer was coated. With the capacity increasing to 12.5 mAh cm^−2^, the DRS was fully filled with Li metal (Supplementary Fig. 4). By contrast, BNS exhibits a fundamentally different mechanism of Li plating. As shown in Fig. 2j, the top of the BNS was preferentially deposited with Li, and the bottom of the BNS remained inert (Fig. 2k, l). The top-view SEM images in Supplementary Fig. 5 further confirm the top-growth for BNS and bottom–up mode for DRS, which were maintained upon cycling as shown in Supplementary Fig. 6. For the typical Cu-foil electrode, Li dendrites were deposited on the top of Cu foil (Supplementary Fig. 7). In contrast to Cu foil and BNS, the DRS exhibited the Li deposition tuned away from the anode/separator interface.

To visualize bottom–up plating more directly, we used an optical microscope to image the plating process. Fig. 2m shows that the silver-gray Li metal was gradually plated from the bottom–up. Fig. 2n, o show optical photographs of the top and bottom surfaces of the Li-plated BNS and DRS with ~5 mAh cm^−2^ capacity (the top surface indicates the side that touches the separator). These images are consistent with the SEM results, demonstrating that DRS preferentially induced Li deposition from bottom up, because the Au layer lowered the nucleation barrier. By contrast, Li was plated in the top region of the BNS because of the Li-ion concentration polarization. To confirm that the plated material was Li and to verify its exact location, we soaked the Li-plated DRS in a CuCl_2_-containing dimethoxyethane solution to displace Li with Cu (see the reaction of Li and CuCl_2_ in Supplementary Fig. 8) because Cu is more readily mapped using the EDX technology than Li^33^. Fig. 2p shows that Cu was only observed in the bottom region, which confirms that the solid phase that filled the bottom pores was metallic Li that was located in the Au-coated regions.

### Coulombic efficiency

**Fig. 3:**
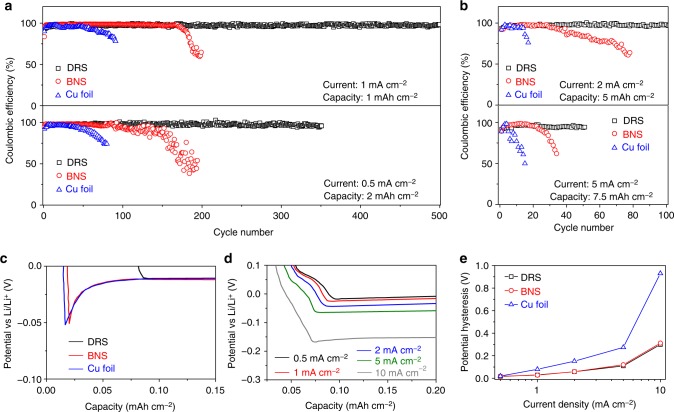
Electrochemical properties of DRS, BNS, and Cu-foil current collectors. **a**, **b** CEs of Li plating/stripping at different current densities and capacities. **c** Nucleation overpotentials for Li plating in the three electrodes at ~0.15 mAcm^−2^. **d** Voltage profiles of Li plating/stripping on a DRS at different current densities. **e** Voltage gap between charge/discharge profiles at different current densities

We measured the electrochemical properties of the Li-plated DRS, as well as Li-plated BNS and Cu foil for comparison (Fig. 3). Li was plated in each cell with capacities ranging from 1 to 7.5 mAh cm^−2^ at varied current densities. These capacity-limited cycling tests demonstrated that the DRS retains a higher CE than the BNS and Cu-foil electrodes over a wide range of operating conditions. In the first few cycles, the CEs of the three samples gradually increased to ~98% (Fig. 3a). However, the CEs of the Cu-foil cells decrease rapidly over the next 80 cycles whereas the CEs of the BNS cells decrease below 50% after ~190 cycles. The DRS retained a CE of ∼98.1% until 500 cycles at 1 mAcm^−2^ in a cell of 1 mAh cm^−2^ and ∼97.0% until 350 cycles at 0.5 mAcm^−2^ in a cell of 2 mAh cm^−2^. The overall CEs of the DRS are far superior to those of BNS and Cu-foil cells. Even at high current densities (up to 5 mAcm^−2^) and high capacities (up to 7.5 mAh cm^−2^), the DRS retains a stable CE for a much greater number of cycles than the BNS and Cu foil (Fig. 3b). High capacities (i.e., high Li loadings) and rates significantly degrade the electrochemical performance of the BNS and Cu-foil anodes. By contrast, the DRS is able to retain a stable CE under these severe test conditions, which is essential for the design of safe, high-energy batteries.

To understand the performance differences among the three anodes, the charge–discharge curves were carefully measured at a low current of ~0.15 mAcm^−2^ to examine the nucleation process. The potential profiles in Fig. 3c show that the BNS and Cu foil need approximately an extra ~40 mV overpotential (as indicated by the dips of curves) for nucleation compared with the DRS. An explanation for the low overpotential is that the Au layer in the DRS reduces the nucleation barrier for Li deposition, resulting from the resembled lattice structures between Li_15_Au_4_ and Li in a Au-Li alloy system^23^. As shown in Fig. 3d, the plating overpotentials for the DRS increase with increasing current density from 0.5 to 10 mAcm^−2^. However, the nucleation overpotentials remain nearly zero, indicating the low-nucleation barrier even at high currents. Fig. 3e presents the potential gap between the plating and stripping plateaus. The Cu-foil anode demands higher overpotentials to drive the Li-plating reactions than the BNS and DRS anodes because Cu foil has a significantly lower surface area for plating than the BNS and DRS. It is interesting to note that the plating overpotentials on BNS are almost the same as those on DRS although their cycling properties differ significantly, which implies that the deposition mode is not truly revealed only using the macroscopic electrical parameters (such as overall current density, overpotential, and resistance) that current safety monitoring techniques rely heavily on. The microstructures of Li metal electrodes play important roles in plating modes and the propensity to short circuit^34^.

### Symmetric cells tests

**Fig. 4:**
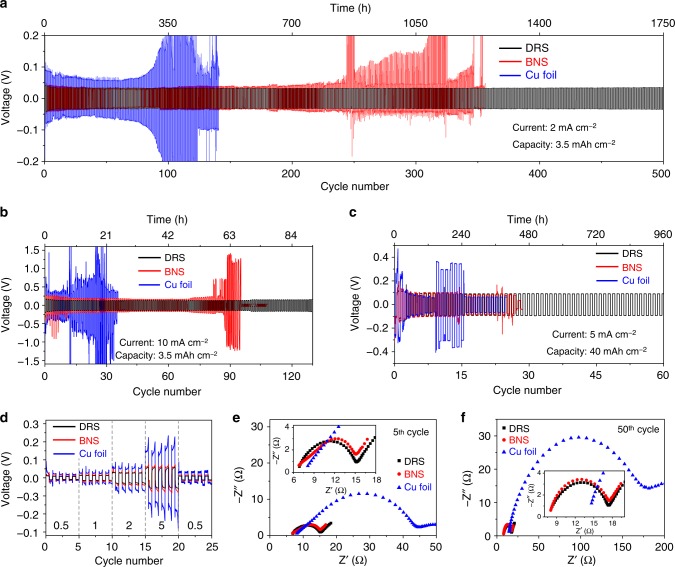
Electrochemical properties of symmetric cells during cycling at high rates and capacities. Voltage profiles of Li plating/stripping with a capacity of 3.5 mAh cm^−2^ at different current densities of **a** 2 mAcm^−2^ and **b** 10 mAcm^−2^. **c** Voltage profiles at an ultrahigh capacity of 40 mAh cm^−2^ at 5 mA cm^−2^. **d** Rate properties of cells with a capacity of 2 mAh cm^−2^ (the unit for each current is mA cm^−2^). Nyquist plots of the three cells at the **e** 5^th^ and **f** 50^th^ cycles, respectively

To eliminate the influence of dissimilar counter electrodes, symmetric cells consisting of two identical electrodes were used to further examine how Li was plated/stripped on the three current collectors (BNS, DRS, and Cu foil, see Supplementary Fig. 9 for their SEM images). Fig. 4a shows the long-term cycling properties of symmetric cells with a capacity of 3.5 mAh cm^−2^ and a current density of 2 mAcm^−2^ (see Supplementary Fig. 10 for enlarged details of voltage curves). The cell consisting of Cu-foil electrodes shows nearly stable voltage profile in the first few cycles. Its voltage starts to oscillate after ~100 cycles (~350 h). The average voltage gaps between the Li plating and stripping profiles significantly increase with the cycle number. For the BNS cell, the voltage gaps slowly increase from ~0.086 to ~0.104 V over ~240 cycles. The voltage profiles start to oscillate rapidly between the 250^th^ and 360^th^ cycles. The random voltage changes for both the BNS and Cu-foil cells can be attributed to the unstable Li/electrolyte interface^35,36^. More specifically, the continuous formation of Li dendrites leads to an excessive passivation layer or SEI^37^, which may increase the total electrical resistance. After a few cycles, there is a sudden voltage drop in the Cu-foil cell, which can be attributed to dendrite-induced short circuits^15,38,39^. By contrast, the voltage profiles of the DRS cell show very consistent plateaus during each cycle. The voltage gaps are generally maintained at ∼0.07 V for more than 500 cycles (1750 h, see Fig. 4a and Supplementary Fig. 11). When the current density increases to 10 mAcm^−2^, the DRS cell can run for 130 cycles, which exceeds the service life of the Cu-foil or BNS cells (Fig. 4b). No short circuits were observed in the DRS cell. At a current density of 30 mAcm^–2^, the DRS cell can still show a certain cycleability (see Supplementary Fig. 12). It implies the excellent mitigation of Li dendrite penetration through the separator. At extremely high capacity of 40 mAh cm^−2^ (Fig. 4c), the DRS cell also cycles much better than the BNS and Cu-foil cells. We summarized some important reports on Li metal anodes in Supplementary Fig. 13. As compared to those previous studies, the DRS demonstrates an excellent electrochemical performances in terms of high current and/or high capacity.

Next, we measured the voltage profiles of the three cells at various current densities (Fig. 4d). The voltage hysteresis of the DRS cell increases only slightly from 18 to 114 mV when the current density increases from 0.5 to 5 mA cm^−2^. By contrast, the Cu-foil cell exhibits a voltage hysteresis of more than ~384 mV at a high current of 5 mAcm^−2^. A peak-like voltage shape at the end of each cycle indicates rapid buildup of overpotential, which may be caused by an unstable SEI or abnormal Li depletion^33,40,41^. The unstable voltage profile of the Cu-foil cell implies the formation of excessive SEI, which also increases the voltage hysteresis at large current densities. It should be noted that the local current densities in the Cu-foil cell are higher than those in the DRS and BNS cells because of the low surface area of the Cu foil, which further contributes to the very large voltage hysteresis (Supplementary Fig. 14).

To substantiate the conclusions above based on the voltage profiles, we examined the resistance change during cycling using electrochemical impedance spectroscopy (EIS). Figure 4e, f show the Nyquist plots of the three cells for the 5^th^ and 50^th^ cycles. The charge-transfer resistance can be estimated from the diameter of the semicircles, which is inversely proportional to the surface area^42^. The large semicircle diameter of the Cu-foil cell indicates a high charge-transfer resistance. The fitted results in Supplementary Table 1 show that the *R*_ct_ of the Cu-foil cell increases more significantly during cycling than those of the DRS and BNS cells. The increase is attributable to the formation of excessive SEI on the Cu-foil cell. It is interesting to note that the EIS results of the DRS and BNS cells are similar at the 5^th^ and 50^th^ cycles. However, after 200 cycles (Supplementary Fig. 15), the DRS cell exhibits much lower impedance than the BNS cell, indicating that the DRS is advantageous for long-term cycling.

### Low-temperature properties

**Fig. 5:**
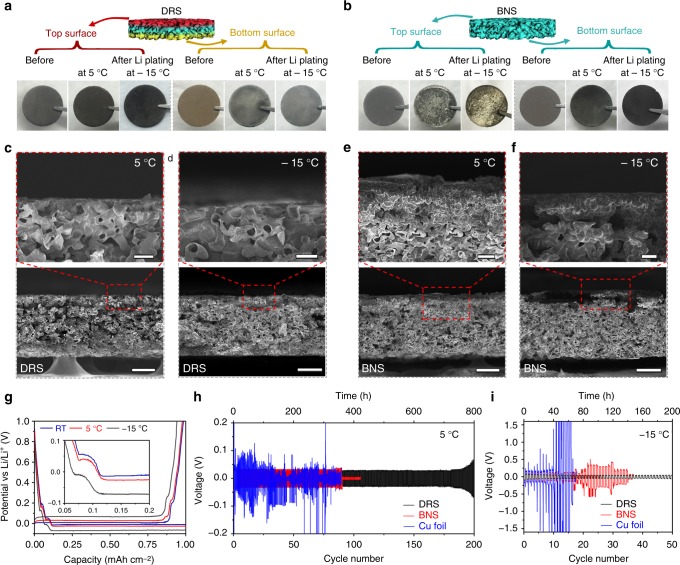
Low-temperature characterizations and electrochemical properties. Optical images of the top and bottom surfaces of **a** DRS and **b** BNS before and after Li plating at 5 °C and −15 °C. **c**–**f** Cross-section SEM images of the top regions at high magnification of (**c**, **d**) DRS and (**e**, **f**) BNS surfaces at 5 °C and −15 °C. **g** Voltage profiles of Li plating/stripping on the DRS at 0.25 mA cm^−2^. Cycling properties of symmetric cells consisting of DRS, BNS, or Cu foil at 0.5 mA cm^−2^ with a limited capacity of 1 mAh cm^−2^ at **h** 5 °C and **i** −15 °C. Scale bars: top images of (**c**−**f**), 5 μm; bottom images of (**c**−**f**), 25 μm

Dendrite-induced short circuits are particularly prevalent at low operating temperature in Li batteries^27,29,30^. Thus, in addition to having established above that the DRS successfully mitigates dendrite formation at room temperature, we investigated its performance at lower temperatures. Figure 5a, b show optical photographs of the top and bottom surfaces of the DRS and BNS, which were plated with Li at 5 °C and −15 °C. The color changes indicate that Li preferentially plated in the bottom region of the DRS at both 5 °C and −15 °C. The SEM images (Fig. 5c, d, and Supplementary Fig. 16) further confirm that, even at low temperatures, the DRS induced Li plating from the bottom to the top. By contrast, Li mainly plated in the top region of the BNS. Especially at −15 °C, the lustrous silver color (Fig. 5b) indicates greater Li deposition in the top region of the BNS than that observed at 5 °C (Fig. 5e, f). As mentioned above, the dendrite formation is generally favored at low temperature. This is because the low mobility of Li ions induces high concentration polarization, which leads to nonuniform Li deposition. Therefore, the bottom–up plating in the DRS indicates that our strategy successfully counteracts the unfavorable influence of concentration polarization on Li plating at low temperature.

The temperature also affects other key electrochemical properties such as overpotential and cycling stability^29–31^. Low temperatures reduce the kinetics of interface charge transfer and increase Li-ion transport resistance in a liquid electrolyte, which results in greater plating overpotentials as indicated in the inset of Fig. 5g. There are still no dips below 0 V on the voltage curves, indicating that the overpotential for nucleation at 5 °C and −15 °C is nearly the same as that at room temperature. Figure 5h shows the cycling properties of the three symmetric cells at 5 °C. The Cu-foil cell has a noisy voltage profile, indicating an unstable SEI. The BNS cell has a relatively stable voltage profile until the 88^th^ cycle, at which the voltage suddenly drops to approximately zero, implying that Li dendrites penetrated the separator and caused a short circuit. The DRS cell maintains a stable voltage profile for more than 180 cycles. The gradually increasing voltage after ~180 cycles indicates the buildup of an SEI without short circuits. Even at −15 °C (Fig. 5i), the DRS cell could cycle 50 times without abrupt voltage changes. By contrast, the BNS and Cu-foil cells show random voltage changes or oscillations after the first few cycles. Although at the increased current density (Supplementary Fig. 17), all three cells degrade significantly as compared to Fig. 5h, i, the DRS cell outperforms both the BNS and Cu-foil cells. Nevertheless, we have demonstrated that the mechanism of tuning Li plating still works at low temperatures.

### Analysis of dendrite growth and cell failure

**Fig. 6:**
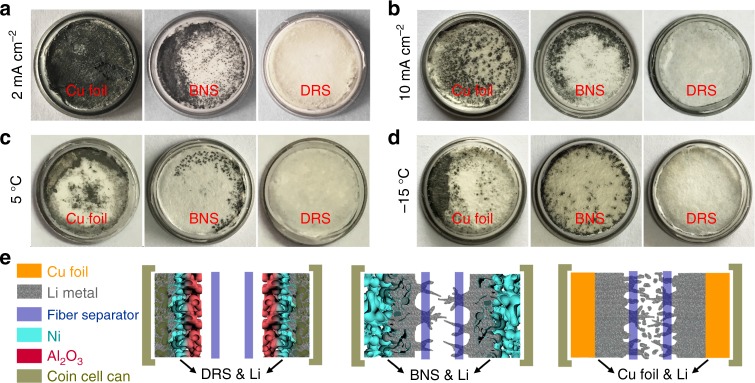
Dendrite detection using symmetric cells including two separators. Optical images of the contact surfaces of two overlapping separators after cycling: **a** at 2 mAcm^−2^ and room temperature (Cu foil for 150 cycles, BNS for 250 cycles, and DRS for 300 cycles), **b** at 10 mA cm^−2^ and room temperature (Cu foil for 50 cycles, BNS for 100 cycles, and DRS for 130 cycles), **c** at 5 °C and 0.5 mA cm^−2^ for 100 cycles and **d** at −15 °C and 0.5 mA cm^−2^ for 50 cycles. **e** Schematic diagrams of different Li-plating modes in cells using Cu foil, BNS, and DRS

To study dendrite growth through the separator and help visualize cell failure under severe operating conditions, we assembled symmetric cells with two stacked glass-fiber separators and conducted a postmortem analysis after cycling at high rates and low temperatures (Fig. 6)^43^. The cycle number for disassembling cells were roughly determined by when the worst cell failed. The good cells were allowed a few more cycles (or until we are able to visualize the performance difference). Figure 6a–d show the inner side of one separator used for dendrite observation. At room temperature, after ~150 cycles at 2 mA cm^−2^ (Fig. 6a), the inner side of the separator in the Cu-foil cell was almost completely covered with black spots (as compared to the image of fresh separator in Supplementary Fig. 18), which indicated that a large quantity of Li dendrites penetrated the separator^42^. In the BNS cell, Li dendrites appeared around the edge of the separator after ~250 cycles. By contrast, for the DRS cell, no dendrites were observed on the separator even after 300 cycles. When the current density was increased to 10 mA cm^−2^ (Fig. 6b), Li dendrites appeared on the separator in the Cu-foil cell after 50 cycles and in the BNS cell after 100 cycles whereas in the DRS, no dendrites were observed after 100 cycles. At the lower temperature (Fig. 6c, d), the separators of the DRS cells also remained clean without observable dendrites (Fig. 6e). These results provide further evidence that the DRS can tune dendrite growth and mitigate dendrite penetration of the separator. This mitigating mechanism of the DRS will improve battery safety in practical applications.

## Discussion

The excellent electrochemical performances of the DRS electrodes can be attributed to the following advantages: (i) The DRS tunes the nucleation thermodynamics to counteract the adverse effects of nonuniform multiphysical fields and induce a safe bottom–up plating mode; (ii) The highly interconnected pores facilitate Li-ion transport and minimize the concentration polarization that results in the preferential Li deposition at the anode/separator interface, which is especially prevalent in high-loading electrodes and/or under harsh operating conditions; (iii) The metallic scaffold maintains good conductivity and accommodates the volume changes during Li-plating and stripping. The strong mechanical properties of the DRS electrodes render the conducting and hosting capabilities marginally affected during charge–discharge cycles; and (iv) The high surface area lowers local current densities and prolongs the Sand’s time, leading to less probable dendrite-induced short circuits (the Sand’s time is a characteristic time at which the ion concentration drops to zero at currents exceeding diffusion limits) because the Sand’s time follows a power law of the current density with a negative exponent.

To further verify and demonstrate the above advantages, we compared DRS with commercial nickel foam (NF). The pore size of NF is around 200 μm ~ 1 mm, which seems too large for high-efficiency Li plating/stripping (Supplementary Fig. 19) unless a more conductive network is constructed inside the large pores. Similar observations were also reported in previous studies^35,39,44,45^. Thus, the small pore size and high porosity make DRS a better scaffold candidate for Li metal anodes. We also applied Au at the bottom of BNS without coating Al_2_O_3_ on the top, only realizing the lithiophilicity gradient, which shows a higher tunability of Li deposition than BNS but lower than DRS (see Supplementary Fig. 20 for details). It indicates that the difference of nucleation overpotentials between the bottom Au and the top Ni was unable to efficiently regulate Li deposition with cycles. With the help of both conductivity and lithiophilicity gradients, DRS outperform Au-coated BNS because Al_2_O_3_ with a conductivity of 10^14^ Ω cm to contrast nickel (6.9 × 10^−6^ Ω cm) enables a large regulating capability to reverse the unfavorable deposition preference caused by multiphysic fields.

In summary, dendrite-induced short circuits are initialized at the anode/separator interface. To prevent preferential deposition of Li at this potentially unsafe interface, we proposed a Li deposition-regulating strategy by using conductivity/lithiophilicity gradients to shift the favorable nucleation spots from the potentially unsafe anode/separator interface to the safe anode bottom. Essentially, the DRS adjusts the local resistances of *R*_e_ and *R*_Li_ in the through-thickness direction of a 3D scaffolded Li-metal anode by electrically passivating the top region of the scaffold and chemically activating its bottom. Because the insulating coating in the top region shuts off the electron conduction in the anode/separator region whereas the Au coating lowers the nucleation overpotentials at the bottom, the unsafe deposition mode was overridden with the bottom–up plating. At spatial and temporal scales, the DRS, to the greatest extent, reduces the probability of the dendrite-induced short circuits. Experimental results demonstrated that the DRS electrode exhibited excellent electrochemical properties at both normal and severe conditions compared to conventional 2D Cu foil and 3D BNS. At last, Au and Al_2_O_3_ were used to demonstrate the strategy. They can be replaced by cheap materials with more functionality for tunable lithiophilicity and conductivity (such as Zn/ZnO, Al, Sn, Si, etc.)^23,25,46^. As the DRS provides an alternative approach to preventing short circuits, which thoroughly differs from previously reported strategies of suppressing dendrites, we believe that combining the DRS and those suppressing strategies can further improve the safety of Li-metal anodes for future high-energy Li-batteries.

## Methods

### Fabrication of 3D DRS

Copper powder (Sinopharm Corp., China) was cast on a graphite plate to form a thin and porous Cu layer. After annealing at 900 °C in forming gas (5% H_2_ and 95% Ar) for ~2 h, the Cu template was peeled off the graphite plate and then inserted into a Ni plating solution (SN-10, Transene Company, USA). Ni was electroplated onto the surface of the Cu template at ~1 mA cm^−2^ for 1–5 h with a Ni plate as the counter electrode. The Cu template in the plated sample was removed in an etchant solution of 0.6 M Na_2_S_2_O_8_, 1.9 M (NH_4_)_2_SO_4_, and 3.5 M NaOH. The resultant Ni scaffold was washed with deionized water and dried in air. The fabrication of nickel scaffold can also be referred to our previous publications^47,48^. The obtained Ni scaffold was washed with deionized water and dried in air. Al_2_O_3_ was directionally deposited into the top region of the Ni scaffold by electron beam evaporation (E-Beam500, Beijing Lako Roya, China). The bottom surface of the Ni scaffold was sputtered with a thin layer of Au (SBC-12, KYKY Corp., China).

### Material and electrochemical characterizations

Morphology observation and elemental mapping were conducted with a Zeiss Ultra 55 field-emission SEM. After electrochemical characterization, the disassembled samples were washed with pure solvent and dried. The cut pieces were transferred to SEM chamber with an Ar-filled box. Optical images were obtained by a Phenom microscope (Pro, Phenom-World). The Cu foil, BNS, and DRS (ϕ15 mm) were assembled in CR2032 coin cells and galvanostatically cycled at various temperatures (25 °C, 5 °C, and −15 °C). The electrolyte was 1 M Li bis (trifluoromethanesulfonyl) imide in a 1:1 volume ratio mixture of 1,3-dioxolane and 1,2-dimethoxyethane with 1 wt% LiNO_3_. Cells were first cycled between 0.01 and 1 V (vs Li/Li^+^) five times to stabilize the SEI^35,49,50^ (see Supplementary Fig. 21 for details). For the fabrication of symmetrical cells, two host electrodes were plated with the same amount of Li and then assembled together to form a symmetric cell. Each cell was usually plated with more Li than the cycling capacities (see Supplementary Fig. 9) according to previous reports^26,35,44,49^. Galvanostatic cycling and EIS were measured using a Land Battery Tester and a VSP potentiostat (Bio-Logic Corp. France).

## Supplementary information

Supplementary Information

## Data Availability

The data that support the findings of this study are available from the corresponding author upon reasonable request.
