# Supplementary material for: Conductivity and lithiophilicity gradients guide lithium deposition to mitigate short circuits
Source: Nat Commun. 2019 Apr 23;10:1896. doi: 10.1038/s41467-019-09932-1 (PMC6478682; doi:10.1038/s41467-019-09932-1)
Supplement: Supplementary file 1 — Supplementary Information [file 41467_2019_9932_MOESM1_ESM.pdf]

Supplementary Information

Conductivity/lithiophilicity gradients guide lithium deposition away from the anode/separator interface to mitigate short circuits

J. Pu et al.

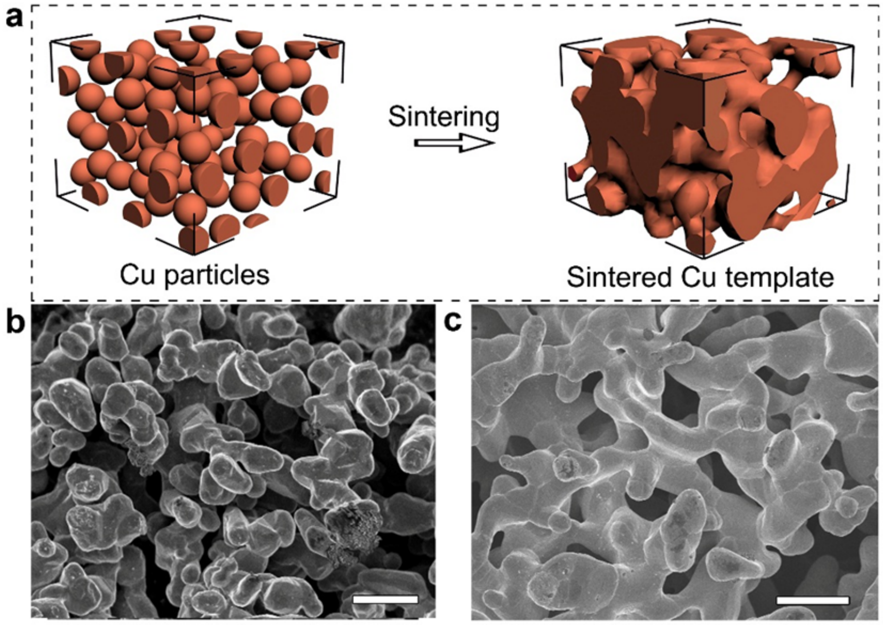

Supplementary Figure 1. Schematic and SEM images of Cu template. (**a**) Schematic illustration of the sintered Cu template from loosely-packed Cu particles. SEM images of (**b**) Cu particles and (**c**) porous Cu template. Scale bars, (**b**, **c**): 5 μm.

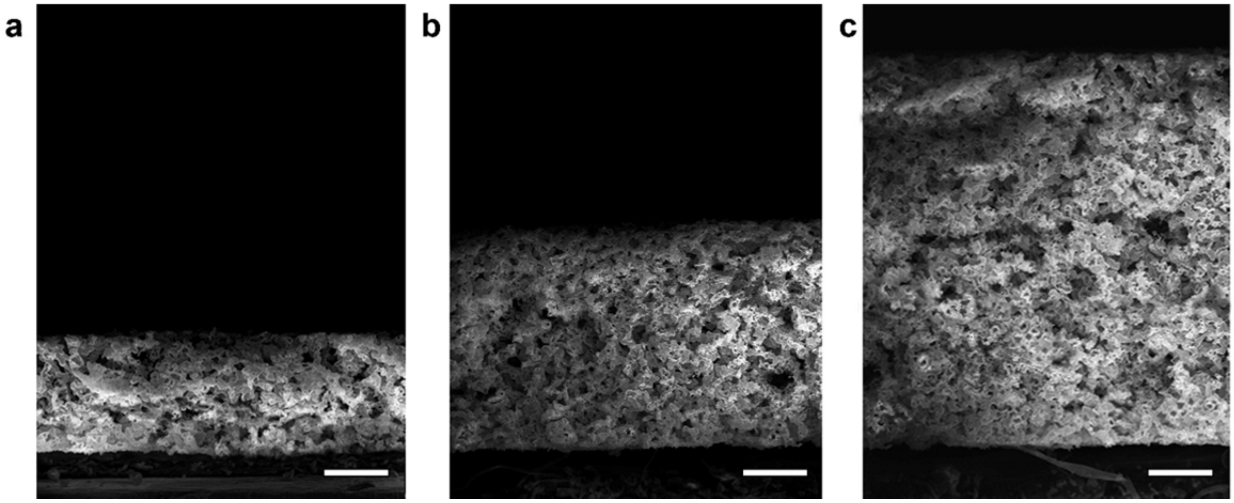

Supplementary Figure 2. SEM images of the porous nickel scaffolds with varied thickness. (**a**−**c**) The thickness is adjusted by using the Cu templates with different thickness (about the detailed scaffold preparation and thickness control, refer to Ref. 47, 48 in the main text). Scale bars, 30 μm.

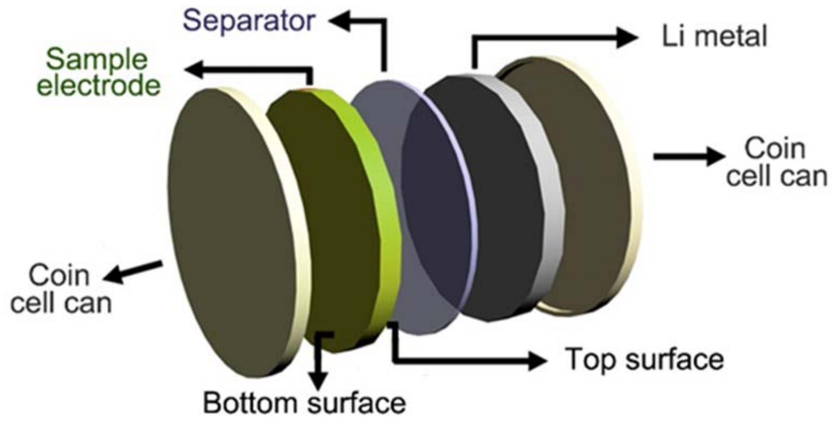

Supplementary Figure 3. Schematic illustration of a cell assembly of metallic Li electrodes with Li as the counter electrode. This figure is used to define the bottom and top surface for accurate description. In the doubled-sided coating cell, the bottom may mean the middle of a Li metal anode.

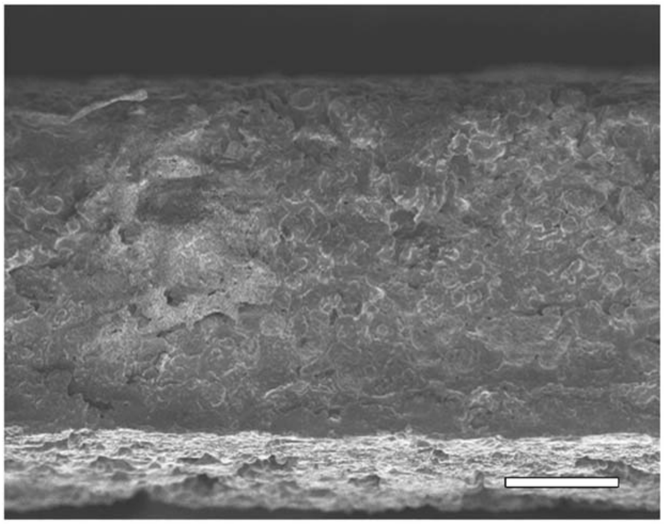

Supplementary Figure 4. SEM image of Li-plated DRS electrode with a loading capacity of 12.5 mAh cm^{-2}. Scale bar, 25 μm.

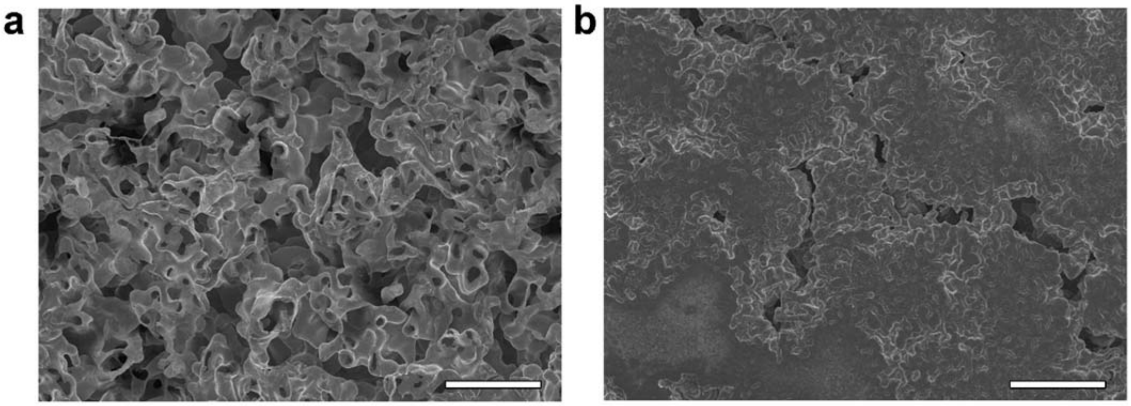

Supplementary Figure 5. SEM images of the electrode top after Li plating with a capacity of 5 mAh cm^{-2}. (**a**) DRS; (**b**) BNS. Scale bars, 20 μm.

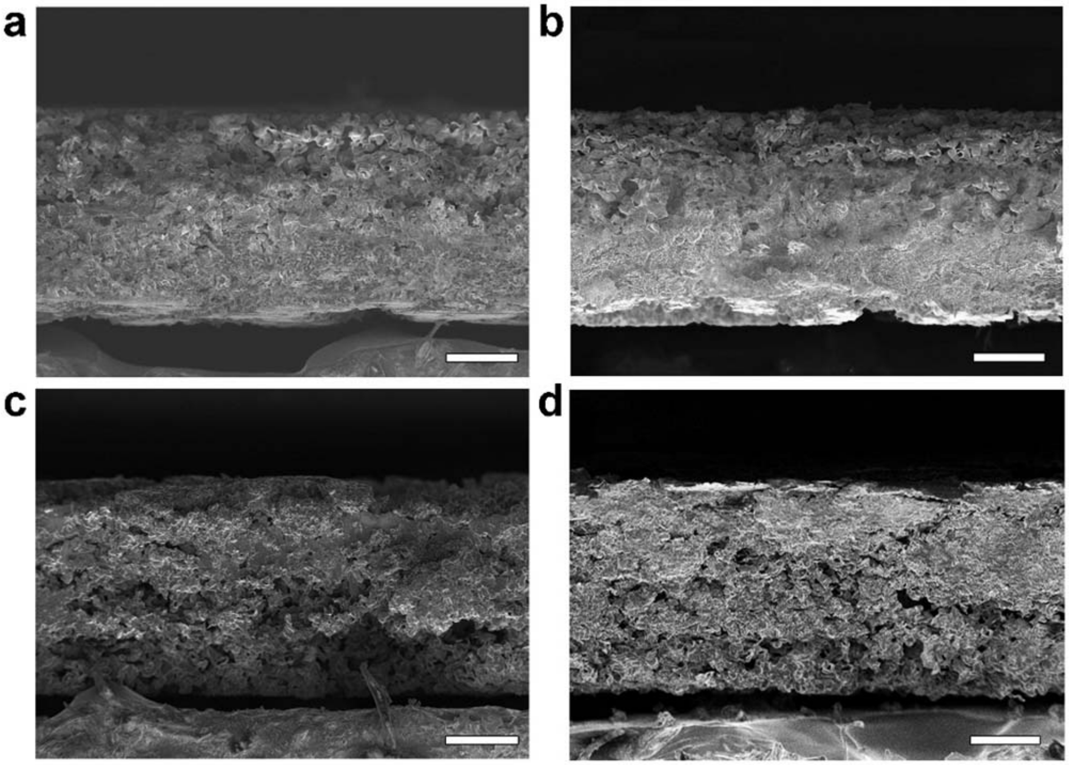

Supplementary Figure 6. Cross-sectional SEM images of the DRS and BNS electrodes with a capacity of 5 mAh cm^{-2} at different cycles. (**a**) DRS at 10th; (**b**) DRS at 30th; (**c**) BNS at 10th; (**d**) BNS at 30th. Scale bars, 25 μm.

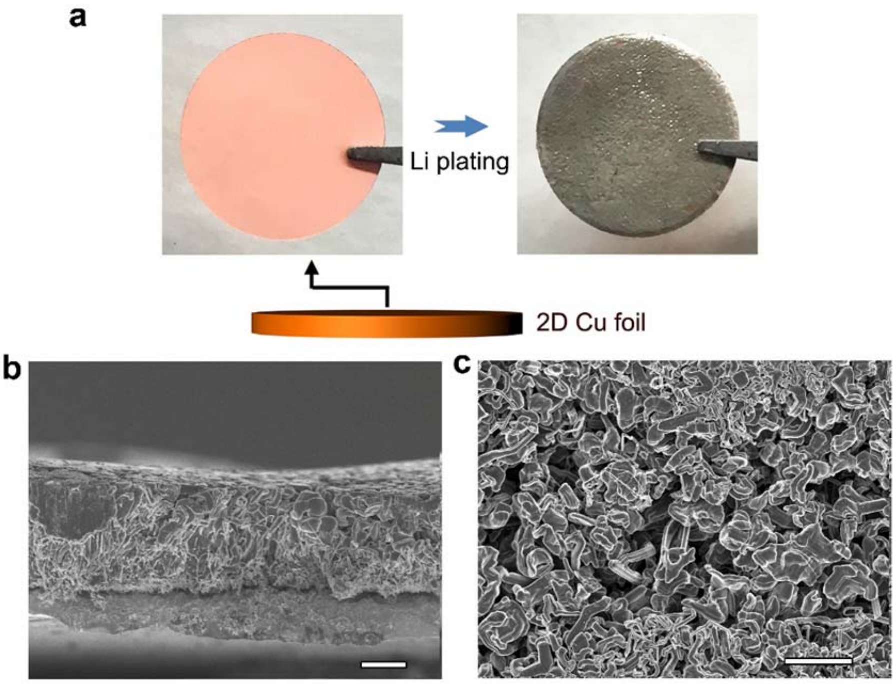

Supplementary Figure 7. Characterization of Li electrodeposition on Cu foil. (**a**) Optical photographs and (**b**, **c**) SEM image of Li-plated Cu foil electrode with 5 mAh cm^{-2} capacity. (**b**) Cross-sectional and (**c**) top-view SEM images. Scale bars, 10 μm.

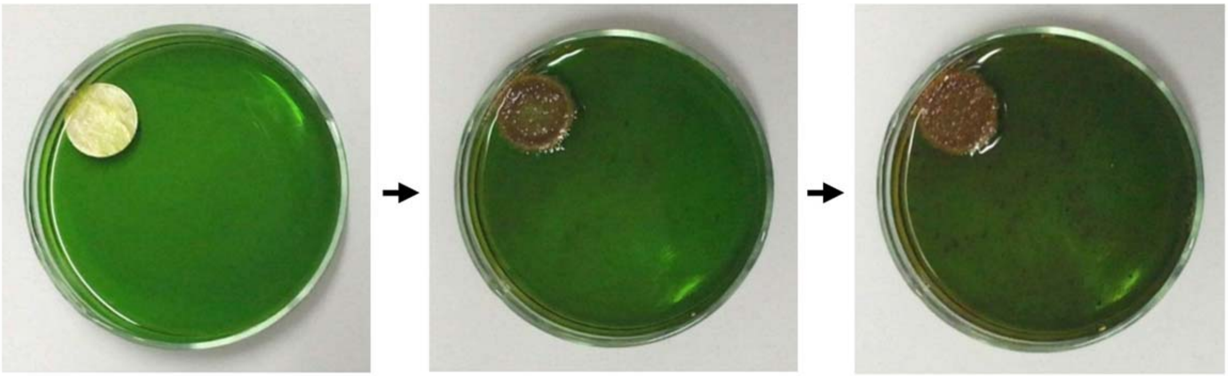

Supplementary Figure 8. Optical photographs of the reaction of Li foil and $CuCl_2$ in dimethoxyethane solution. This figure is to show the *in-situ* replacement reaction of Li for Cu.

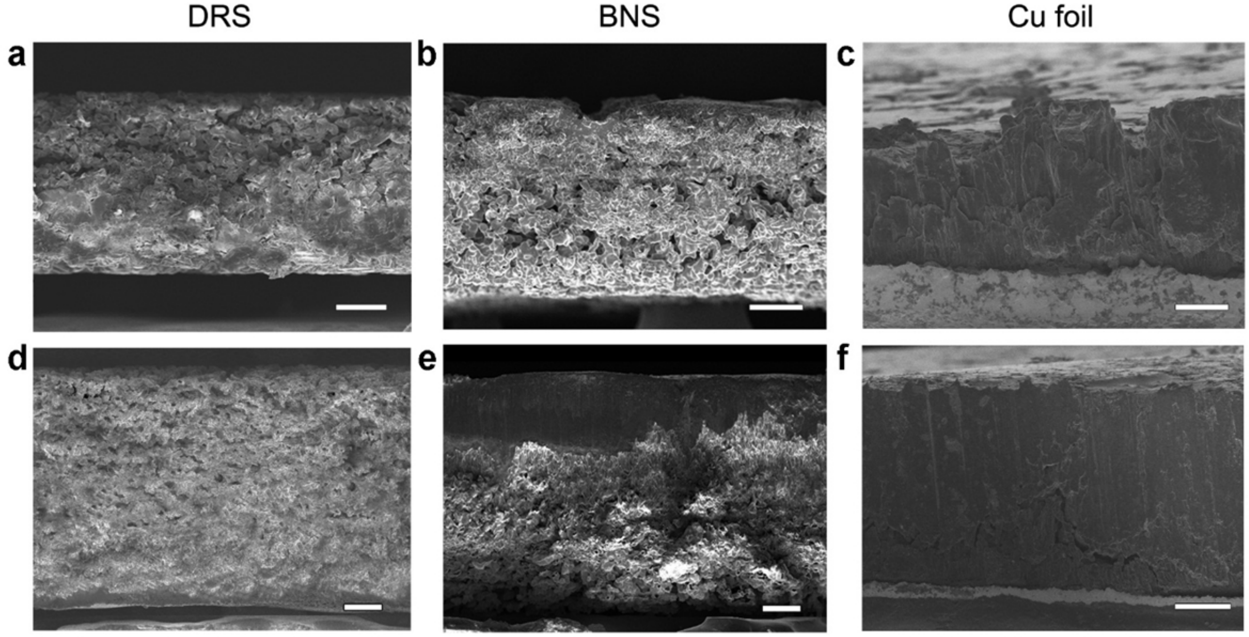

Supplementary Figure 9. Cross-sectional SEM images of Li-loaded electrodes for symmetric cells. (**a**, **d**) DRS, (**b**, **e**) BNS, and (**c**, **f**) Cu foil. Li is loaded with (**a**−**c**) 7 and (d-f) 40 mAh cm^{-2} on each electrodes, respectively. The cycling capacity of their symmetric cells is controlled at (**a**−**c**) 3.5 and (**d**−**f**) 40 mAh cm^{-2}, respectively, as shown in Fig. 4a−c. Scale bars, (**a**, **b**): 25 μm; (**c**): 10 μm; (**d**−**f**): 50 μm.

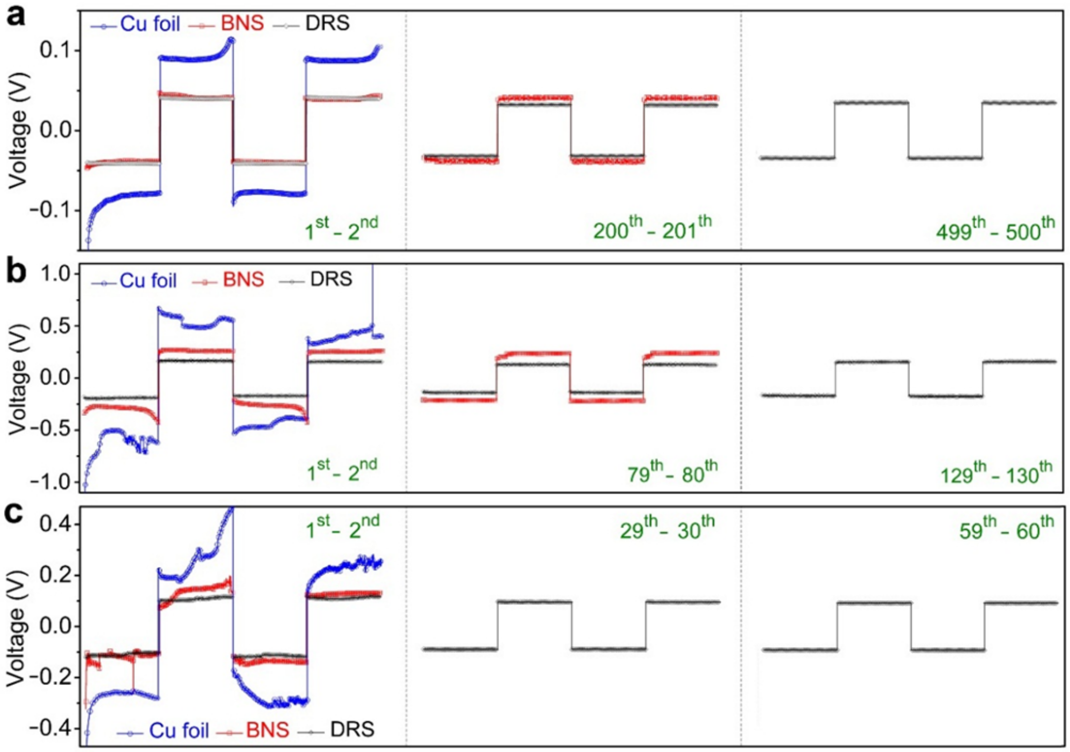

Supplementary Figure 10. Enlarged voltage curves of symmetric cells using DRS, BNS, and Cu-foil electrodes with varied cycling capacities and current densities at serval typical cycle numbers. (**a**) 3.5 mAh cm^{-2} and 2 mA cm^{-2}, (**b**) 3.5 mAh cm^{-2} and 10 mA cm^{-2}, (**c**) 40 mAh cm^{-2} and 5 mA cm^{-2}.

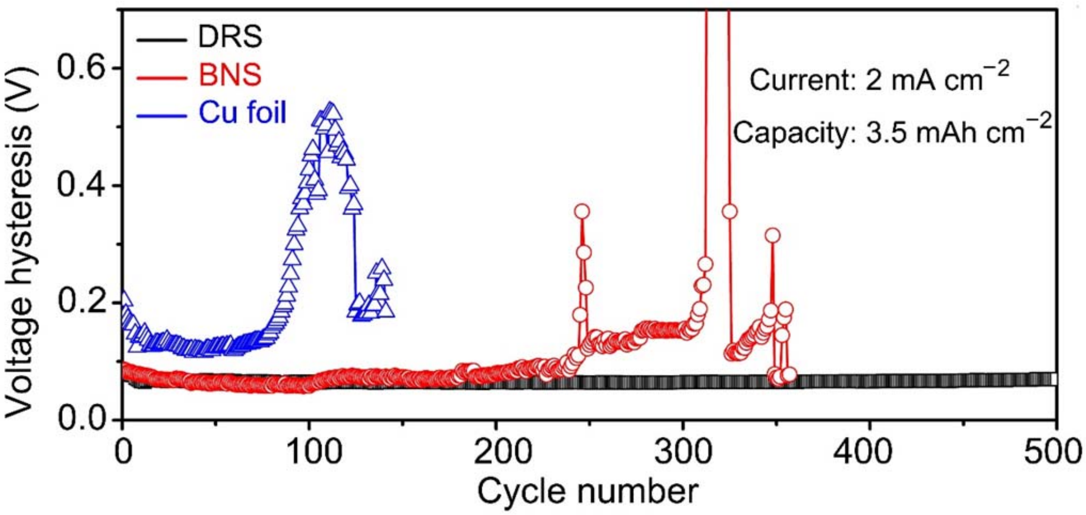

Supplementary Figure 11. Voltage hysteresis with the cycle number, which corresponds to Fig. 4a.

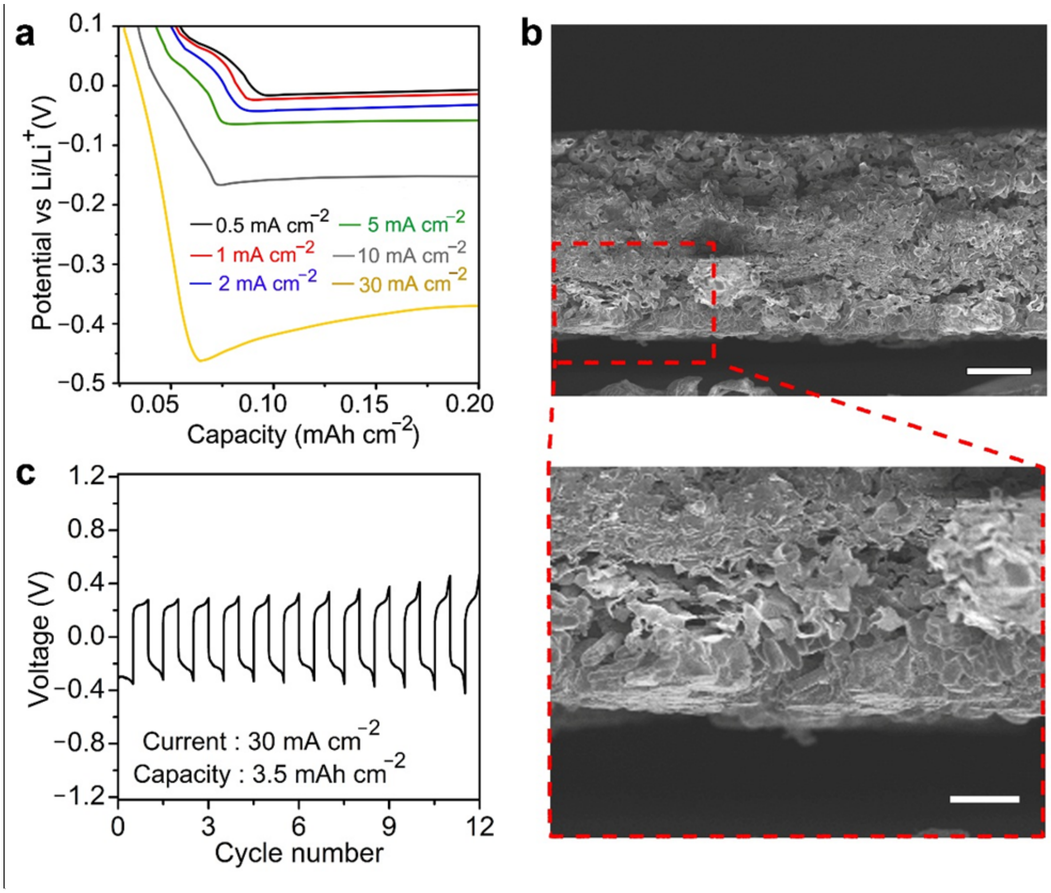

Supplementary Figure 12. SEM and electrochemical characterization of electrode at high current density. (**a**) Voltage curves of the DRS electrodes (*vs* Li metal anodes). It mainly shows the difference of the nucleation and plating overpotentials at varied current densities. (**b**) Cross-sectional SEM images of a DRS electrode with a Li loading of 7 mAh cm^{-2}. Scale bars, top: 25 µm; bottom: 10 µm. (**c**) Cycling voltage profiles of a symmetrical cell using two DRS electrodes with a loading capacity of 7 mAh cm^{-2} and cycling capacity of 3.5 mAh cm^{-2}. (see Supplementary note 1)

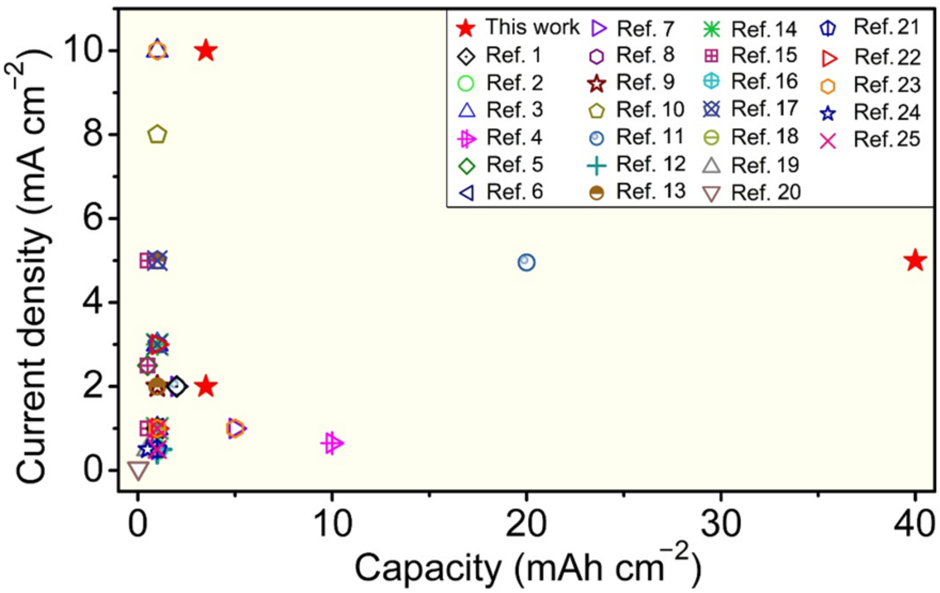

Supplementary Figure 13. Comparison of the areal capacity and current density of Li metal anodes in this work and previous literatures[1–25].

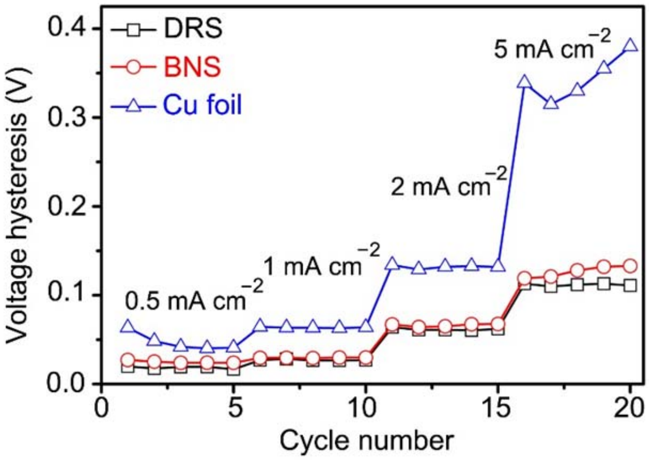

Supplementary Figure 14. Average voltage gap between Li plating and stripping profiles at varied current densities. It corresponds to the rate properties of three symmetric cells in Fig. 4d.

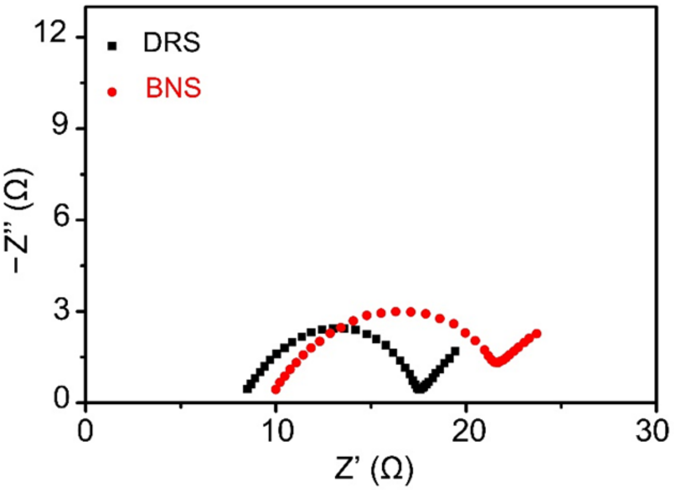

Supplementary Figure 15. Nyquist plots at the 200th cycle for the BNS and DRS.

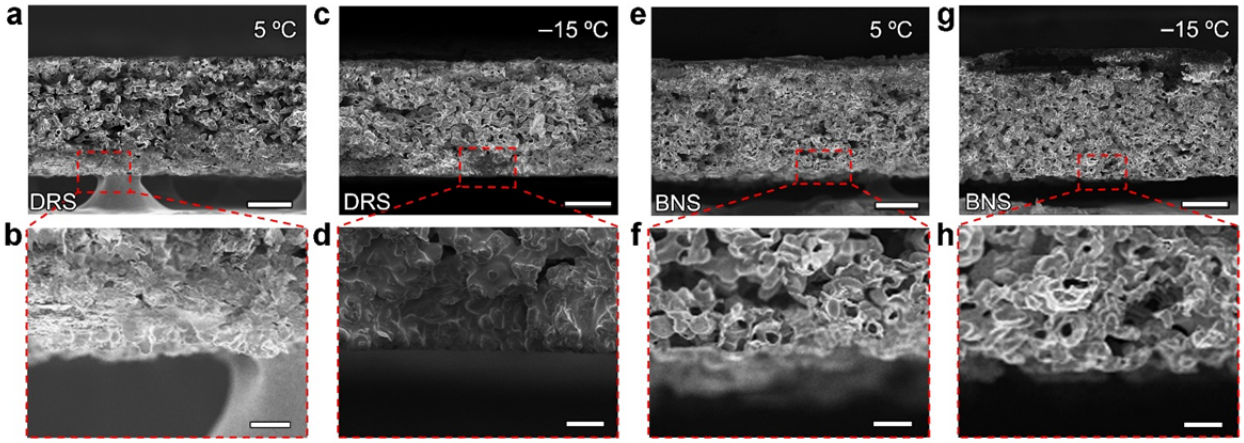

Supplementary Figure 16. Cross-section SEM images of the top regions at high magnification of the DRS and BNS bottom at 5 and −15 °C. The electrode of DRS (**a**, **b**) at 5 °C and (**c**, **d**) at −15 °C. The electrode of BNS (**e**, **f**) at 5 °C and (**g**, **h**) at −15 °C. Scale bars, (**a**, **c**, **e**, **g**): 5 μm; (**b**, **d**, **f**, **h**): 25 μm.

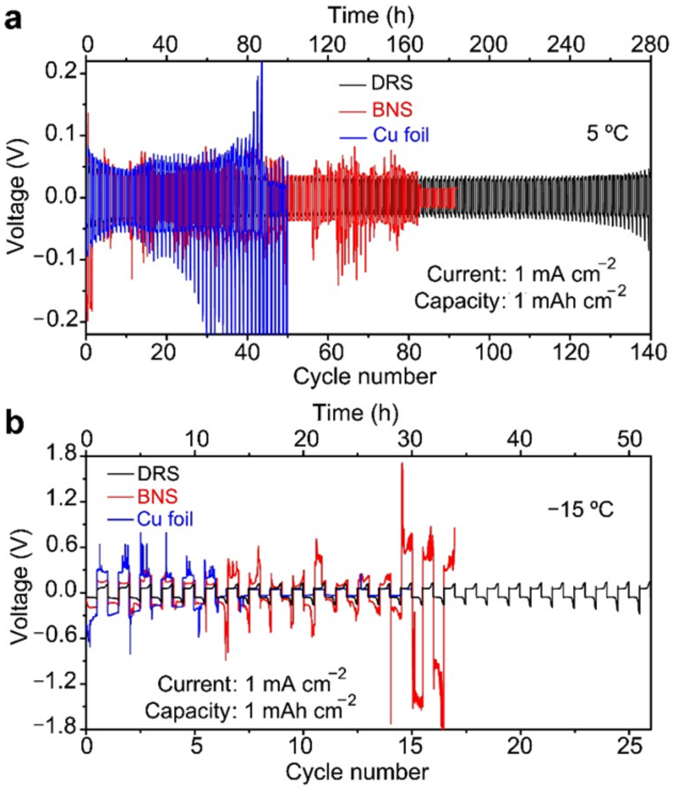

Supplementary Figure 17. Cycling properties of symmetric cells consisting at 1 mA cm^{-2} with a limited capacity of 1 mAh cm^{-2} at low temperatures. (**a**) 5 °C and (**b**) −15 °C.

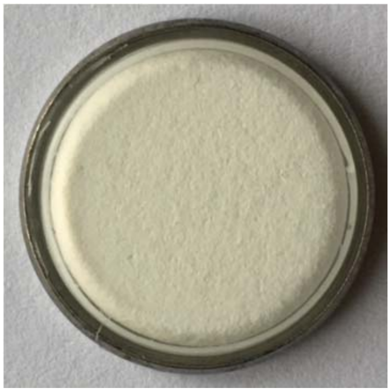

Supplementary Figure 18. Optical photograph of the glass-fiber separator that was used for the dendrite detection tests. To visualize the dendrite penetration, the image can be used for the comparison with the images in Fig. 6.

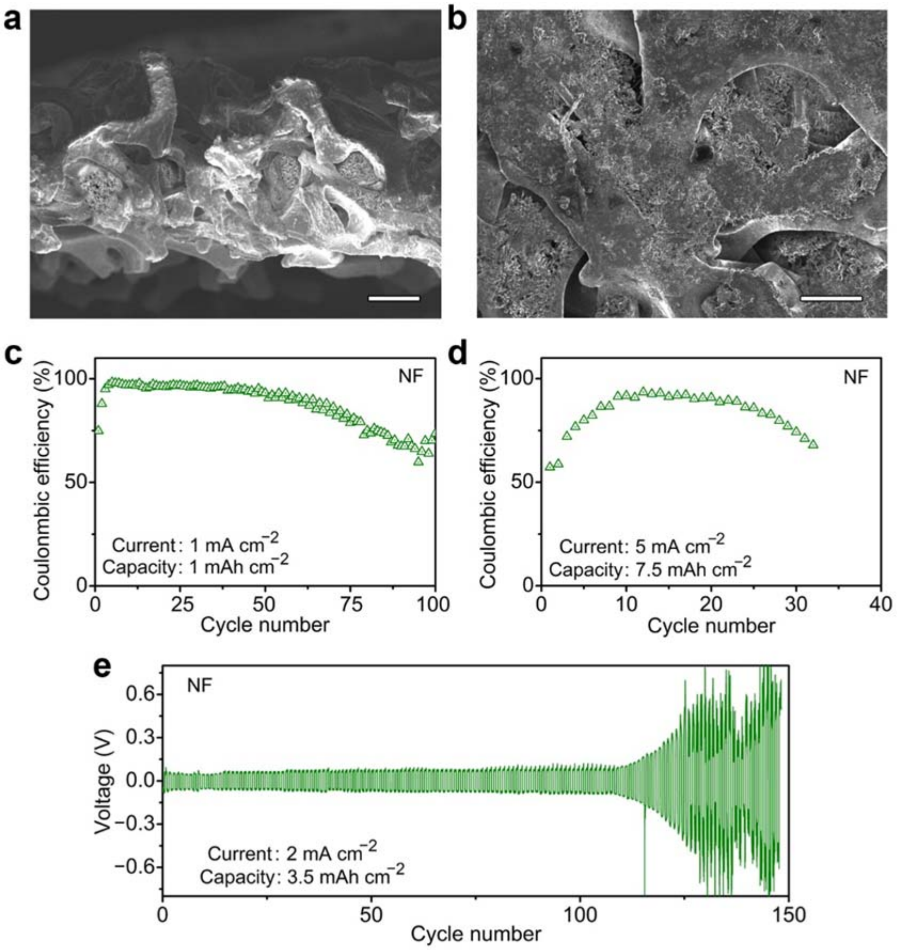

Supplementary Figure 19. SEM and electrochemical characterization of NF electrode. (**a**) Cross-sectional and (**b**) top-view SEM images of the Li-plated NF with a capacity of 7 mAh cm^{-2}. Scale bars, (**a**) 100 µm; (**b**) 50 µm. (**c**, **d**) Coulombic efficiency of commercial NF with varied capacity and at current densities. (**e**) Cycling voltage curves of symmetric cells using two identical electrodes with a Li loading of 7 mAh cm^{-2} and cycling capacity of 3.5 mAh cm^{-2}. (see Supplementary note 2)

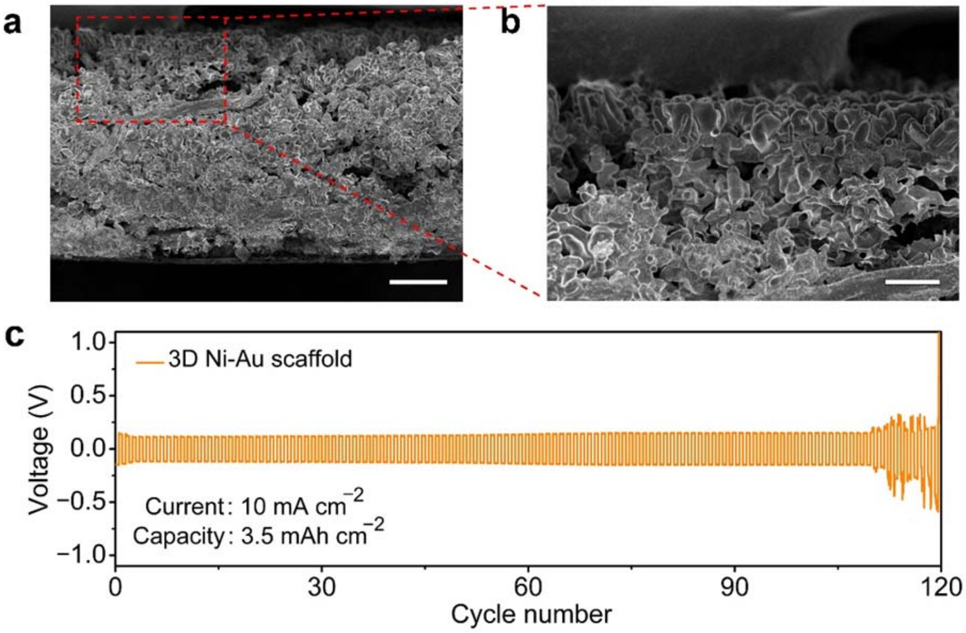

Supplementary Figure 20. SEM and electrochemical characterization of Au-coated BNS electrode. (**a**, **b**) Cross-sectional SEM images of Au-coated BNS upon cycling at 2 mA cm^{-2}. Scale bars, (**a**) 25 μm; (**b**) 10 μm. (**c**) Voltage curves of symmetric cells using two identical Au-coated BNS with a Li loading of 7 mAh cm^{-2}. The cycling capacity was set to 3.5 mAh cm^{-2}. (see Supplementary note 3)

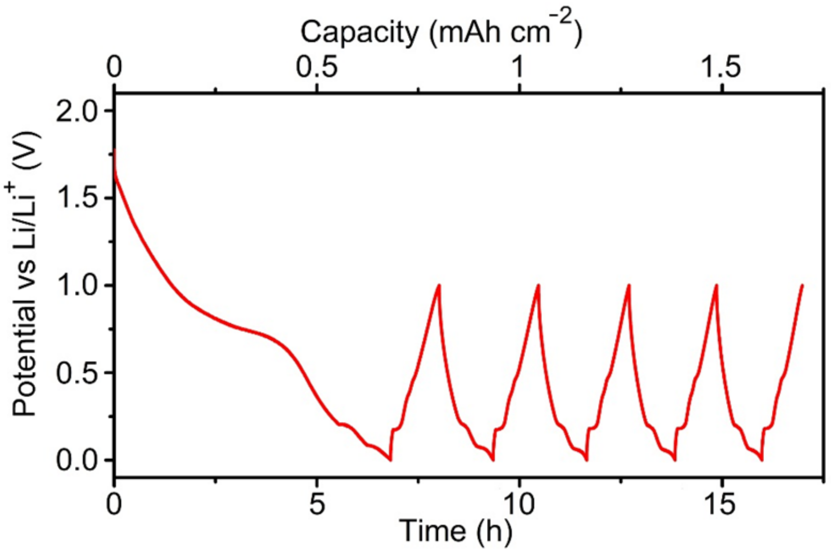

Supplementary Figure 21. Typical voltage profile during pre-lithiation process for DRS. (see Supplementary note 4)

Supplementary Tables

Supplementary Table 1. Impedance parameters from the EIS spectra of the three symmetric cells.

Sample	R_s (Ω)			$Rs + R_{ct}$ (Ω)		
	5th	50th	200th	5th	50th	200th
DRS	7	8	9	15	17	17
BNS	7	8	10	15	17	22
Cu foil	8	15	--	45	178	--

Supplementary Notes

Supplementary Note 1

As shown in Supplementary Fig. 12, a nucleation overpotential of about 100 mV appears at 30 mA cm^{-2} as compared to the zero-barrier curves at low current densities. It is inferred that the nucleation overpotentials may increase faster with the increase of current densities than the plating overpotentials, or nucleation occurs on both gold (bottom) and nickel (middle) exposed regions at such a high current density. The SEM image in Supplementary Fig. 12b shows that at such a high current density, Li was plated into both the bottom and middle regions. Supplementary Fig. 12c shows the voltage curves of a symmetric cell with a cycling capacity of 3.5 mAh cm^{-2} at 30 mA cm^{-2}. The cell can be cycled 12 times. It can be seen that the stripping/plating voltages at the end of each cycle increase rapidly.

Supplementary Note 2

Supplementary Fig. 19c,d shows the Coulombic efficiency (CE) upon cycling and voltage curves of a symmetrical cell using two identical commercial nickel foam (NF) electrodes. As compared to DRS, NF is cycleable at low loading and low current densities. At a relatively high capacity and current density, the NF electrode degrades rapidly, showing much low CEs. The symmetric cell shows a relatively stable voltage profiles until 60 cycles. Since that, the voltage gaps of the NF cell increase more rapidly than those of BNS and DRS cells, but slower than the Cu foil cell. It is understandable that the high surface area of NF lowers the local current densities on the internal surface, improving the cycleability of Li metal anodes based on NF. To explain why the NF cell decays much rapidly than the BNS and DRS cells, we disassembled the cycled cells and observed the morphology of plated Li (Supplementary Fig. 19a,b). It was found that Li was deposited onto the skeleton of NF. Ultra-large pore size and high porosity led to inhomogeneous deposition of lithium metal. There are some big voids, which were not filled with Li. The void is around several tens of micrometers. Such an inhomogeneous deposition results from the lack of dense electronic network in spite of the conductive nickel skeleton.

Supplementary Note 3

From Supplementary Fig. 20, because gold can guide the Li nucleation, Li also prefers to deposit from bottom up in a Au-coated host. However, Supplementary Fig. 20a,b shows that at high rates, there are some part of Li plated onto the bare top of the Au-coated BNS. The voltage curves in Supplementary Fig. 20c demonstrate that Au-coated BNS can be cycled for 110 times in the symmetric cell until the voltage oscillation. The cycleability of Au-coated BNS is higher than bare BNS but lower than that of DRS. Therefore, it leads us to conclude that a single strategy works for regulating Li deposition and however, the tunability is limited as compared to the systemic strategy.

Supplementary Note 4

In Supplementary Fig. 21, the current collectors were first pre-lithiated by cycling at about 0.1 mA cm^{-2} from 0.01 to 1 V (*vs* Li/Li^{+}) for five cycles. In the first lithiation curve, the plateau at about 0.7 V may be ascribed to the reaction with surface oxides and the formation of SEI. The two mini-plateaus (~0.2 and ~0.1 V) indicate the formation of Li-Au alloys with a capacity of ~0.1 mAh cm^{-2}, which was negligible as compared to the overall capacity of Li metal when they are assembled as a lithium metal anode.

Supplementary References

1. Cheng, X.-B. et al. Nanodiamonds suppress the growth of lithium dendrites. *Nat. Commun.* **8**, 336 (2017).
2. Cui, J., Yao, S. S., Ihsan-Ul-Haq, M., Wu, J. X. & Kim, J.-K. Correlation between Li plating behavior and surface characteristics of carbon matrix toward stable Li metal anodes. *Adv. Energy Mater.* **9**, 1802777 (2019).
3. Wang, A. X. et al. Horizontal centripetal plating in the patterned voids of Li/graphene composites for stable lithium-metal anodes. *Chem* **4**, 2192−2200 (2018).
4. Ren, F. H. et al. Pseudocapacitance induced uniform plating/stripping of Li metal anode in vertical graphene nanowalls. *Adv. Funct. Mater.* **28**, 1805638 (2018).
5. Yan, C. et al. An armored mixed conductor interphase on a dendrite-free lithium-metal anode. *Adv. Mater.* **30**, 1804461 (2018).
6. Wang, Z. H. et al. Conducting polymer paper-derived mesoporous 3D N-doped carbon current collectors for Na and Li metal anodes: A combined experimental and theoretical study. *J. Phys. Chem. C* **122**, 23352−23363 (2018).
7. Chen, T. et al. Ionic liquid-immobilized polymer gel electrolyte with self-healing capability, high ionic conductivity and heat resistance for dendrite-free lithium metal batteries. *Nano Energy* **54**, 17−25 (2018).
8. Liao, K. M. et al. Developing a "water-defendable" and "dendrite-free" lithium-metal anode using a simple and promising $GeCl_4$ pretreatment method. *Adv. Mater.* **30**, 1705711 (2018).
9. Xie, J. et al. Engineering stable interfaces for three-dimensional lithium metal anodes. *Sci. Adv.* **4**, eaat5168 (2018).

10. Qin, L. Q. et al. Fabrication of lithiophilic copper foam with interfacial modulation toward high-rate lithium metal anodes. *ACS Appl. Mater. Interfaces* **10**, 27764−27770 (2018).

11. Shi, Q. W., Zhong, Y. R., Wu, M., Wang, H. Z. & Wang, H. L. High-capacity rechargeable batteries based on deeply cyclable lithium metal anodes. *Proc. Natl Acad. Sci. USA* **115**, 5676−5680 (2018).

12. Kim, P. J., Kim, K. & Pol, V. G. Uniform metal-ion flux through interface-modified membrane for highly stable metal batteries. *Electrochim. Acta* **283**, 517−527 (2018).

13. Deng, W., Zhu, W. H., Zhou, X. F., Peng, X. Q. & Liu, Z. P. Highly reversible Li plating confined in three-dimensional interconnected microchannels toward high-rate and stable metallic lithium anodes. *ACS Appl. Mater. Interfaces* **10**, 20387−20395 (2018).

14. Zhang, Y. et al. 3D wettable framework for dendrite-free alkali metal anodes. *Adv. Energy Mater.* **8**, 1800635 (2018).

15. Yan, C. et al. Dual-layered film protected lithium metal anode to enable dendrite-free lithium deposition. *Adv. Mater.* **30**, 1707629 (2018).

16. Ma, L. B. et al. Nanoporous and lyophilic battery separator from regenerated eggshell membrane with effective suppression of dendritic lithium growth. *Energy Storage Mater.* **14**, 258−266 (2018).

17. Zhang, C. Y., et al. Incorporating ionic paths into 3D conducting scaffolds for high volumetric and areal capacity, high rate lithium-metal anodes. *Adv. Mater.* **30**, 1801328 (2018).

18. Xie, K. Y. et al. Dual functionalities of carbon nanotube films for dendrite-free and high energy-high power lithium-sulfur batteries. *ACS Appl. Mater. Interfaces* **9**, 4605−4613 (2017).

19. Liu, Y. Y. et al. Transforming from planar to three-dimensional lithium with flowable interphase for solid lithium metal batteries. *Sci. Adv.* **3**, eaao0713 (2017).

20. Wang, C. W. et al. Universal soldering of lithium and sodium alloys on various substrates for batteries. *Adv. Energy Mater.* **8**, 1701963 (2018).

21. Yang, C. P. et al. Ultrafne silver nanoparticles for seeded lithium deposition toward stable lithium metal anode. *Adv. Mater.* **29**, 1702714 (2017).

22. Lang, J. L. et al. Surface graphited carbon scaffold enables simple and scalable fabrication of 3D composite lithium metal anode. *J. Mater. Chem. A* **5**, 19168−19174 (2017).

23. Wang, L. Y. et al. ZnO/carbon framework derived from metal-organic frameworks as a stable host for lithium metal anodes. *Energy Storage Mater.* **11**, 191−196 (2018).

24. Hou, G. M. et al. Dendrite-free Li metal anode enabled by a 3D free-standing lithiophilic nitrogen-enriched carbon sponge. *J. Power Sources* **386**, 77−84 (2018).

25. Ren, F. H. et al. Over-potential induced Li/Na filtrated depositions using stacked graphene coating on copper scaffold. *Energy Storage Mater.* **16**, 364−373 (2019).
